# Evolutionary Process of Deep-Sea *Bathymodiolus* Mussels

**DOI:** 10.1371/journal.pone.0010363

**Published:** 2010-04-27

**Authors:** Jun-Ichi Miyazaki, Leonardo de Oliveira Martins, Yuko Fujita, Hiroto Matsumoto, Yoshihiro Fujiwara

**Affiliations:** 1 Faculty of Education and Human Sciences, University of Yamanashi, Kofu, Yamanashi, Japan; 2 Department of Biochemistry, Genetics and Immunology, University of Vigo, Vigo, Spain; 3 Institute of Biological Sciences, University of Tsukuba, Tsukuba, Ibaraki, Japan; 4 Research Program for Marine Biology and Ecology, Japan Agency for Marine-Earth Science and Technology (JAMSTEC), Yokosuka, Kanagawa, Japan; Northern Fisheries Centre, Australia

## Abstract

**Background:**

Since the discovery of deep-sea chemosynthesis-based communities, much work has been done to clarify their organismal and environmental aspects. However, major topics remain to be resolved, including when and how organisms invade and adapt to deep-sea environments; whether strategies for invasion and adaptation are shared by different taxa or unique to each taxon; how organisms extend their distribution and diversity; and how they become isolated to speciate in continuous waters. Deep-sea mussels are one of the dominant organisms in chemosynthesis-based communities, thus investigations of their origin and evolution contribute to resolving questions about life in those communities.

**Methodology/Principal Finding:**

We investigated worldwide phylogenetic relationships of deep-sea *Bathymodiolus* mussels and their mytilid relatives by analyzing nucleotide sequences of the mitochondrial cytochrome *c* oxidase subunit I (COI) and NADH dehydrogenase subunit 4 (ND4) genes. Phylogenetic analysis of the concatenated sequence data showed that mussels of the subfamily Bathymodiolinae from vents and seeps were divided into four groups, and that mussels of the subfamily Modiolinae from sunken wood and whale carcasses assumed the outgroup position and shallow-water modioline mussels were positioned more distantly to the bathymodioline mussels. We provisionally hypothesized the evolutionary history of *Bathymodilolus* mussels by estimating evolutionary time under a relaxed molecular clock model. Diversification of bathymodioline mussels was initiated in the early Miocene, and subsequently diversification of the groups occurred in the early to middle Miocene.

**Conclusions/Significance:**

The phylogenetic relationships support the “Evolutionary stepping stone hypothesis,” in which mytilid ancestors exploited sunken wood and whale carcasses in their progressive adaptation to deep-sea environments. This hypothesis is also supported by the evolutionary transition of symbiosis in that nutritional adaptation to the deep sea proceeded from extracellular to intracellular symbiotic states in whale carcasses. The estimated evolutionary time suggests that the mytilid ancestors were able to exploit whales during adaptation to the deep sea.

## Introduction

Deep-sea mussels of the genus *Bathymodiolus* (Mytilidae, Bathymodiolinae) are one of the dominant macroorganisms in chemosynthesis-based communities in hydrothermal vents on spreading ridges and back-arc basins and in cold-water seeps along subduction zones. Since the original description of the genus [1], 22 *Bathymodiolus* species have been described [2]–[13], and their biogeographic distributions are as follows. There are: 1) 14 Pacific species, *B. japonicus* Hashimoto & Okutani 1994, *B. platifrons* Hashimoto & Okutani 1994, *B. septemdierum* Hashimoto & Okutani 1994, *B. hirtus* Okutani et al. 2004, *B. securiformis* Okutani et al. 2004, *B. aduloides* Hashimoto & Okutani 1994, *B. taiwanensis* Cosel 2008, *B. brevior* Cosel et al. 1994, *B. elongates* Cosel et al. 1994, *B. tangaroa* Cosel & Marshall 2003, *B. manusensis* Hashimoto & Furuta 2007, *B. edisonensis* Cosel and Janssen 2008, and *B. anteumbonatus* Cosel and Janssen 2008 from the West Pacific and *B. thermophilus* Kenk & Wilson, 1985 from the East Pacific; 2) seven Atlantic species, *B. childressi* Gustafson et al. 1998, *B. heckerae* Gustafson et al. 1998, and *B. brooksi* Gustafson et al. 1998 from the West Atlantic, *B. azoricus* Cosel & Comtet 1999 and *B. puteoserpentis* Cosel et al. 1994 from the Mid-Atlantic Ridge, and the trans-Atlantic *B. mauritanicus* Cosel 2002 and *B. boomerang* Cosel & Ole 1998; and 3) one Indian Ocean species, *B. marisindicus* Hashimoto 2001. Two species of the genus *Gigantidas* from the West Pacific, *G. horikoshii* Hashimoto &Yamane 2005 and *G. gladius* Cosel & Marshall 2003, and one species of the genus *Tamu* from the Atlantic, *T. fisheri* Gustafson et al. 1998, belong to the subfamily Bathymodiolinae [5], [9], [14]. Active exploration of new localities and careful surveys of known localities suggests the existence of many cryptic species. The species diversity is very high in the West Pacific compared with other areas, and thus the origin of the bathymodioline mussels seems to be located in the West Pacific. However, the mismatch distributions of the West Pacific *B. septemdierum* and *B. brevior* and the Indian Ocean *B. marisindicus* suggest that the Southern Central Indian Ridge of the Indian Ocean might be the more ancient residence rather than the Izu-Ogasawara Island-arc and the North Fuji Basin of the West Pacific, if periods from formation to expansion of their populations were not significantly different among them [15].

**Figure 1:**
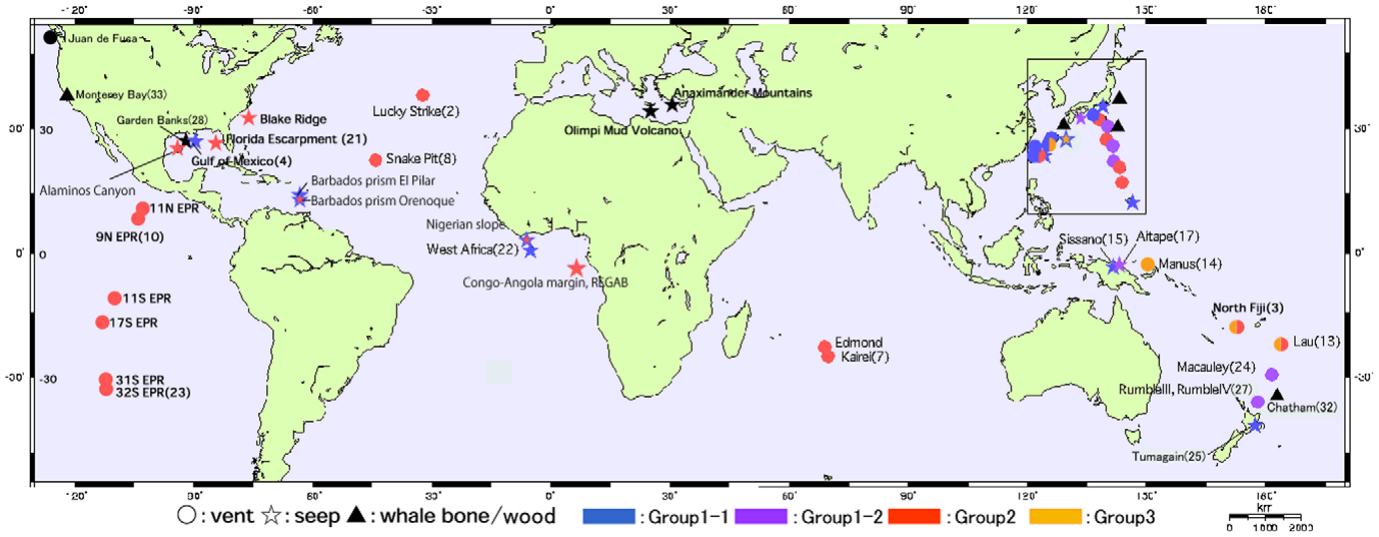
The sampling sites for deep-sea *Bathymodiolus* mussels and their relatives used in this study. Refer to Table 2 for details of the sampling sites. ○, hydrothermal vent; •, cold-water seep; ▪, wood/whale bone; ▴, shallow.

**Figure 2:**
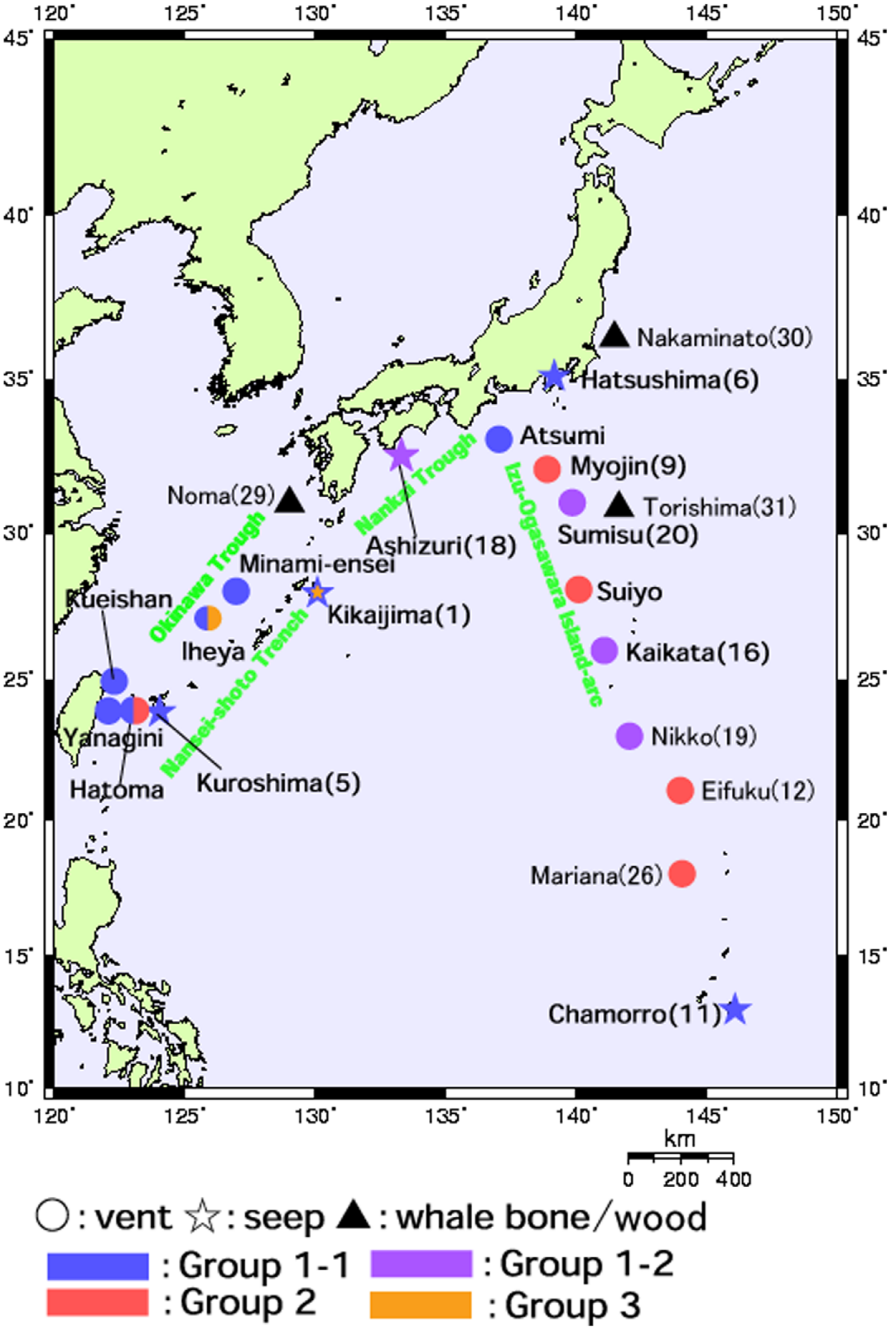
The sampling sites for deep-sea *Bathymodiolus* mussels and their relatives in Japanese waters. The boxed region in Fig. 1 is enlarged. Refer to Table 2 for details of the sampling sites. ○, hydrothermal vent; •, cold-water seep; ▪, wood/whale bone; ▴, shallow.

In Japanese waters (Figs. 1 and 2), six *Bathymodiolus* and one *Gigantidas* species have steady residences as evidenced by a stable, constant supply of their propagules [3, 10. 14]. Some species possibly have transient residences through incident, leaky supply of propagules as mentioned below. *Bathymodiolus japonicus* and *B. platifrons* are distributed in seeps in Sagami Bay and vents of the Okinawa Trough, which are separated by approximately 1,500 km. *Bathymodiolus aduloides* is distributed in seeps in Sagami Bay, the Nankai Trough, and the subduction zone of the Nansei-shoto Trench and vents of the Okinawa Trough. However, our genetic analyses have not confirmed its existence in the Nankai Trough. The Nankai Trough is situated between Sagami Bay and the Okinawa Trough. There appear to be some barriers to gene flow between the Nankai Trough and Sagami Bay and between the Nankai Trough and Okinawa Trough. Only one specimen, identified genetically as *B. aduloides*, has been obtained so far from vents in the Izu-Ogasawara Island-arc. The three species of *Bathymodiolus* mussels can exploit both seeps and vents as habitats. No significant genetic differentiation was discernible between seep and vent populations of *B. platifrons* [15]. Our studies also suggested a genetic similarity between seep and vent populations of *B. japonicus* [16], indicating the high adaptability of these species to deep-sea environments, albeit the seemingly large environmental differences between seeps and vents. *Bathymodiolus septemdierum* is distributed in vents in the Izu-Ogasawara Island-arc, but not in Sagami Bay, which is approximately 500 km from the Myojin Knoll and Suiyo Seamount in the Izu-Ogasawara Island-arc. Only one specimen, identified genetically as *B. septemdierum*, has been obtained so far from the Okinawa Trough. There are relatively large obstacles to gene flow between Sagami Bay and the Izu-Ogasawara Island-arc. Our genetic studies suggested that this species was conspecific to *B. brevior* and might possibly be conspecific to *B. marisindicus* [15]. If this is the case, this species has the widest habitat range among *Bathymodiolus* species from Japanese waters southeastward to the North Fuji Basin in the West Pacific Ocean and southwestward to the Kairei Field in the Indian Ocean. The two remaining species, *B. hirtus* and *B. securiformis*, are distributed in seeps of the subduction zone of the Nansei-shoto Trench. The latter is also distributed in seeps in the Nankai Trough. *Gigantidas horikoshii* is distributed in vents in the Izu-Ogasawara Island-arc. One specimen from Sagami Bay has been identified as Sissano *B.* sp. 1, which resides mainly in Sissano in the West Pacific Ocean.

Organisms initially invading the deep sea encounter serious difficulties and must alter their feeding strategies to overcome poor nutrition and acquire tolerance to high pressure and cold seawater. Furthermore, organisms in vents and seeps must establish symbiosis with chemosynthetic bacteria as an effective feeding strategy and tolerance to toxic H_2_S. The “Evolutionary stepping stone hypothesis” has been proposed, in which the ancestors of bathymodioline mussels exploited sunken wood and whale carcasses in their progressive adaptation to deep-sea environments [17], [18]. Further studies are required to elucidate the origin and adaptive process of bathymodioline mussels as a representative of organisms in chemosynthesis-based communities. Our previous research suggested an evolutionary transition from shallow water to vent/seep sites via sunken wood/whale carcass sites and supported the hypothesis [15], [19], although deeper branching was poorly supported. However, the process did not occur in a single, unidirectional manner. Our research also suggested independent invasion into vents and seeps and reversion into whale carcass sites from vent or seep sites in the mytilid lineages.

Only some *Bathymodiolus* species from limited areas were the subjects of earlier molecular phylogenetic studies [18], [20]–[22]. Subsequently, using updated databases, molecular phylogenetics searched for the phylogeny of about 10 species [16], [23], [24], and mytilid relatives from whale carcasses and wood were included to trace the origins of *Bathymodiolus* mussels [25], [26]. In our previous studies [15], [19], we sequenced the mitochondrial genes of more than 15 nominal and cryptic species, and showed that mussels in the subfamily Bathymodiolinae comprised four groups. The first group (Group1) includes seven West Pacific and Atlantic *Bathymodiolus* species (Group 1-1) and two West Pacific *Gigantidas* species (Group 1-2). The group includes members of the *B. childressi* clade (*B. childressi*, *B. mauritanicus*, *B. platifrons*, *B. hirtus*, *B. anteumbonatus*, *B. japonicus*, *B. tangaroa*. *B. securiformis*, *B. edisonensis*, and two *Gigantidas* species) based on morphological traits [13]. The second group (Group 2) includes six or seven *Bathymodiolus* species, which are subdivided into three subclusters consisting of the Indo-West Pacific, Atlantic, and East Pacific species, respectively, with the exception of *B. brooksi* that diverges basally to the subclusters. Group 2 includes members of Cosel's *B. thermophilus* clade (*B. thermophilus* and East Pacific *B.* sp., *B. brevior*, *B. azoricus*, *B. elongates*, *B. puteoserpentis*, *B. septemdierum*, *B. boomerang*, *B. heckerae*, and *B. brooksi*). The third group (Group 3) includes two West Pacific *Bathymodiolus* species, and the fourth group includes only one species, *T. fisheri*. The group includes members of Cosel's *B. aduloides* clade (*B. aduloides* and *B. manusensis*). Our studies also showed that the subfamily Bathymodiolinae and the genus *Bathymodiolus* were not monophyletic, suggesting the need to reevaluate the classification.

In the present study, we investigated worldwide phylogenetic relationships of *Bathymodiolus* mussels and their mytilid relatives by analyzing concatenated sequences of the mitochondrial cytochrome *c* oxidase subunit I (COI) and NADH dehydrogenase subunit 4 (ND4) genes. We also investigated the evolutionary processes of *Bathymodiolus* mussels by estimating evolutionary divergence times with variable rates over time.

## Results

### Phylogenetic relationships of *Bathymodiolus* mussels and their relatives

**Figure 3:**
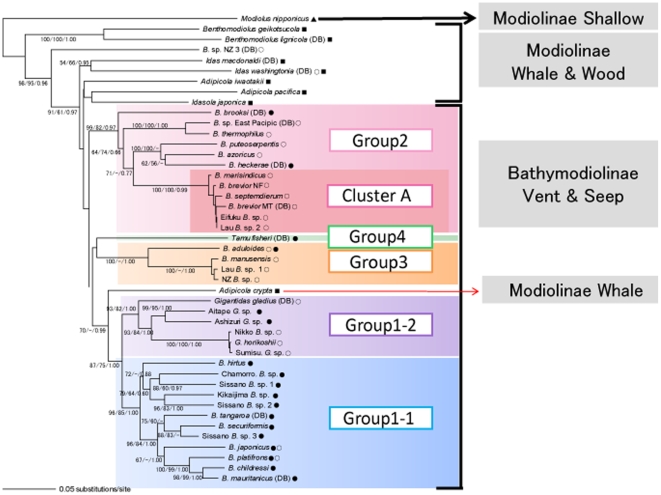
Phylogenetic relationships of deep-sea *Bathymodiolus* mussels and their relatives based on the 401-bp COI and 423-bp ND4 sequences. The NJ tree was constructed based on the genetic distances calculated according to Kimura's two-parameter method using *Modiolus nipponicus* as an outgroup species. The MP and Bayesian trees presented essentially the same topology as the NJ tree. Only the NJ (left) and MP (middle) bootstrap values >50% and Bayesian posterior probabilities (right) >0.50 are specified. The scale bar indicates 0.01 substitutions per site. See Table 1 for abbreviations of *Bathymodiolus* mussels and their relatives. ○, hydrothermal vent; •, cold-water seep; ▪, wood/whale bone; ▴, shallow.

Mussels of the subfamily Bathymodiolinae were divided into four groups (Fig. 3). The first group (Group 1) was subdivided into two subgroups. One subgroup (Group 1-1) consisted of seven nominal species, *B. hirtus*, *B. japonicus*, *B. platifrons*, and *B. securiformis* from Japanese waters, *B. tangaroa* from the West Pacific, *B. mauritanicus* and *B. childressi* from the Atlantic, and five unidentified (not morphologically examined) *Bathymodiolus*-related mussels from Sissano (Sissano *B.* sp. 1, *B.* sp. 2, and *B.* sp. 3), the Chamorro Seamount (Chamorro *B.* sp.), and off Kikaijima Island (Kikaijima *B.* sp.) in the West Pacific. All the members so far examined in Group 1-1 (6 nominal species and Sissano *B*. sp.) have methanotrophic endosymbionts [27]–[28]. Group 1-2 included two nominal species, *G. horikoshii* and *G. gladius*, and four unidentified *Gigantidas*-related mussels from the Nikko Seamount (Nikko *G.* sp.), Sumisu Caldera (Sumisu *G.* sp.), Aitape (Aitape *G.* sp.), and off Ashizuri Cape (Ashizuri *G.* sp.) in the West Pacific. The former two unidentified mussels are likely to be conspecific with *G. horikoshii* because of their genetic similarity. The species has thioautotrophic endosymbionts (the data will be published elsewhere).

The second group (Group 2) consisted of eight nominal and one undescribed (morphologically examined but not yet described) *Bathymodiolus* species. This group was subdivided into three subclusters including the Indo-West Pacific *B. septemdierum*, *B. brevior*, and *B. marisindicus*, Atlantic *B. azoricus*, *B. puteoserpentis*, and *B. heckerae*, and East Pacific *B. thermophilus* and undescribed species (East Pacific *B.* sp.), with the exception of the Atlantic *B. brooksi*, which diverged basally to the three clusters. *Bathymodiolus septemdierum*, *B. brevior*, and *B. marisindicus* comprised the closely related species group (Cluster A [16]). We showed in our previous study that a high gene flow occurred between *B. septemdierum* and *B. brevior* and that the gene flow between *B. marisindicus* and *B. septemdierum* or *B. brevior* was low but not negligible, although their habitats are approximately 5,000–10,000 km apart [15]. Mussels from the Lau Basin (Lau *B.* sp.1) and Eifuku Seamount (Eifuku *B.* sp.) included in the cluster are likely to be conspecific to *B. septemdierum* because of their genetic similarity. Four species in Group 2, *B. septemdierum*, *B. brevior*, *B. marisindicus*, and *B. thermophilus*, contain solely thioautotrophs, and *B. puteoserpentis*, *B. azoricus*, *B. heckerae*, and *B. brooksi* harbor both thioautotrophs and methanotrophs [27], [29]–[35].

The third group (Group 3) consisted of two nominal species, *B. aduloides* and *B. manusensis*, restricted to the West Pacific. Mussels from the Lau Basin (Lau *B.* sp. 2) and off New Zealand (NZ *B.* sp.) are likely to be conspecific with *B. manusensis* because of their genetic similarity. Species in this group contain thioautotrophic endosymbionts [36]. The above three groups, two subgroups, and three subclusters were well supported. The fourth group (Group 4) consisted only of *T. fisheri*. Groups 3 and 4 were allied and in turn they were allied with Group 1, but the relationships were poorly supported.

Mussels of the subfamily Modiolinae from sunken wood and whale carcasses assumed the outgroup position to the bathymodioline mussels from vents and seeps, with the exception of *Adipicola crypta* (Dall et al. 1938) from whale carcasses. The species was allied with Group 1 with marginal support. The cluster including vent/seep bathymodioline mussels and wood/whale modioline mussels was well supported. Mytilid mussels flourishing in shallow water were positioned more distantly to the vent/seep bathymodioline mussels. Although only the modioline *Modiolus nipponicus* (Oyama 1950) was included in the present tree, modioline *Modiolus modiolus* (Linnaeous 1758) and three species of the subfamily Mytilinae were also positioned, as was *M. nipponicus* in our previous studies [15]. The unity of *Bathymodiolus* mussels was not supported, because two species of *Gigantidas* in the Bathymodiolinae and *A. crypta* in the Modiolinae perturbed the unity.

### Estimation of divergence time

**Figure 4:**
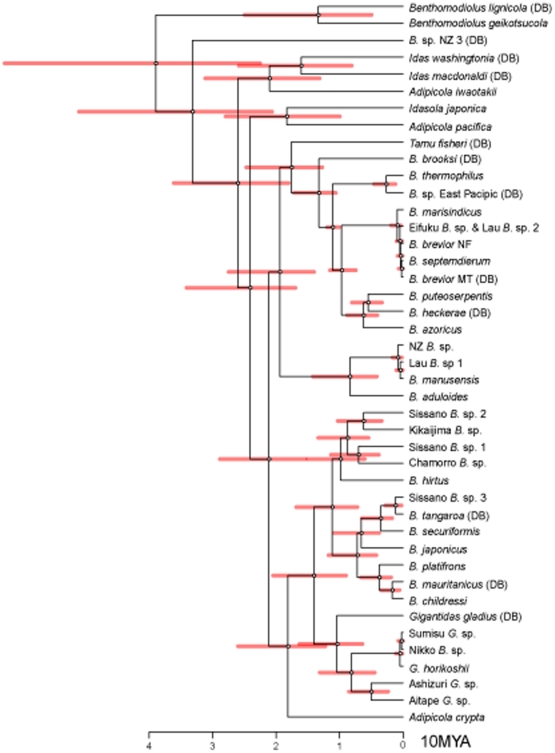
Posterior distribution of evolutionary divergence times. Phylogenetic relationships of deep-sea *Bathymodiolus* mussels based the concatenated 401-bp COI and 423-bp ND4 sequences. The ML tree was constructed using *Modiolus nipponicus* as an outgroup species. The red lines represent 95% credibility intervals of sampled values. See Table 2 for abbreviations of *Bathymodiolus* mussels.

Estimating divergence time is useful to reconstruct evolutionary history. The mean evolutionary rate of mitochondrial DNA has been estimated to be 1∼2% per million years [37]. However, the application of a molecular clock is problematic in some cases, because the rate constancy of molecular evolution is a prerequisite [38], [39]. Our preliminary study showed that the rate of molecular evolution varied among lineages of *Bathymodiolus* mussels, and thus we adopted Thorne and Kishino's approach (see [40] for details of the application of this approach). We show estimates of evolutionary time on the ML tree (Fig. 4). For calibration, we used reference time associated with the split between the Atlantic and East Pacific subclusters (12 to 10 MYA) in Group 2. Our results (Table 1) showed that diversification of bathymodioline mussels initiated in the early Miocene (about 20 MYA). Subsequently, Groups 1 to 3 started differentiating in the early to middle Miocene (about 19 to 14 MYA).

**Table 1:** Estimated evolutionary time (t).

	t (MYA)
Onset of diversification of the subfmaily Bathumodiolinae	21.1±3.6
Split of Groups 2 and 3	19.4±3.4
Split of Groups 1-1 and 1-2	14.1±3.0
Split of *B. brooksi* and the other members in Group2	13.3±1.7
Split of East Pacific subcluster from the common ancestor of Indo-West Pacfic and Atlantic subclusters in Group 2	(12∼10)^a^
Onset of diversification in *Bathymodiolus* spp. of Group 1-1	11.2±2.5
Onset of diversification in *Gigantidas* spp. of Group 1-2	10.4±2.5
Split of *B. hirtus* from the common ancestor of Sissano *B*. spp. 1 and 2, Kikaijima *B.* sp., and Chamorro *B*. sp. in Group 1-1	9.8±2.3
Split between Indo-West Pacfic and Atlantic subclusters in Group 2	9.7±1.0
Split of *B. aduloides* and *B. manusensis* in Group 3	8.4±2.6
Split of *B. japonicus* from the common ancestor of *B. securiformis*, *B. tangaroa*, and Sissano *B.* sp. 3 in Group 1-1	6.6±1.8
Onset of diversification of Atlantic species in Group 2	6.2±1.2
Split of *B. platifrons* from the common ancestor of *B. mauritanicus* and *B. childressi* in Group 1-1	3.8±1.2
Split of *B. securiformis* from the common ancestor of *B. tangaroa* and Sissano *B.* sp. 3 in Group 1-1	3.5±1.5
Split of *B. thermophilus* and East Pacific sp. in Group 2	2.6±0.9
Onset of diversification in Cluster A of Group 2	0.9±0.4

a reference time.

## Discussion

### Phylogenetic relationships of *Bathymodiolus* mussels and their relatives

Bathymodioline species from vents and seeps were divided into four well-supported groups (Fig. 3). Together they comprised the poorly-supported bathymodioline cluster. Concatenated sequence data, however, provided better resolution of the phylogeny of *Bathymodiolus* mussels and their relatives than those derived from single COI [19] or ND4 [15] sequence data, although some OTUs could not be used because of the lack of sequence data on either gene. Modioline species from sunken wood and whale carcasses assumed the outgroup position to the bathymodioline mussels, with the exception of *A. crypta* from whale carcasses. Mytilid species from shallow water such as *M. nipponicus* were positioned more distantly to the bathymodioline mussels. The results support the “Evolutionary stepping stone hypothesis,” which advocates adaptive progress of deep-sea organisms from shallow-water to vent/seep sites via wood/whale carcass sites [17], [18].

**Figure 5:**
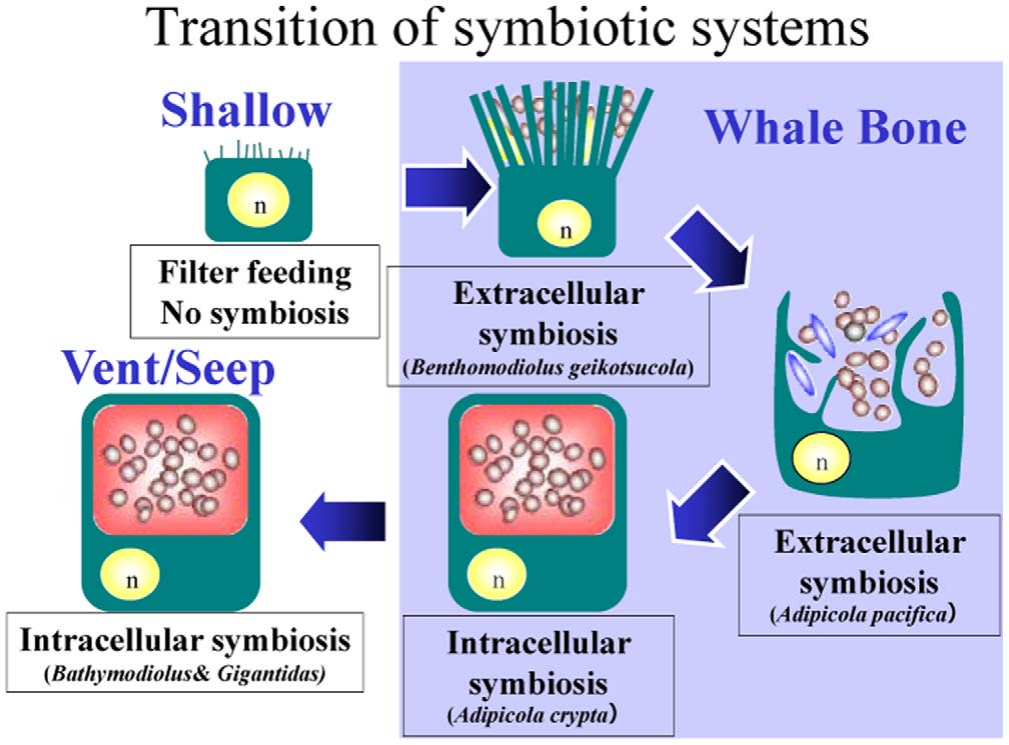
Schematic representation of evolutionary symbiostic transition. Mytilid mussels from shallow water with no symbionts; *Benthomodiolus geikotsucola* from whale carcasses haboring extracellular symbionts trapped by microvilli of the host cells; *Adipicola pacifica* from whale carcasses haboring extracellular symbionts enclosed by the protrudent host cell membrane; *A. crypta* from whale carcasses haboring intracellular symbionts; *Bathymodiolus* mussels with intracellular symbionts.

Three modioline species, *Benthomodiolus geikotsucola* Okutani & Miyazaki 2007, *A. pacifica* (Dall et al. 1938), and *A. crypta*, were obtained from whale carcasses in Japanese waters, and the epidermal cells of their gills harbored thioautotrophic bacterial symbionts [15], although no mytilid mussels haboring symbionts have previously been reported from shallow water. As shown schematically in Fig. 5, *Benthomodiolus geikotsucola* from naturally sunken Bryde's whale carcasses at the Torishima Seamount (approximately 4,000 m in depth) had extracellular symbionts trapped by microvilli of the host cells (the data will be published elsewhere). *Adipicola pacifica* and *A. crypta* inhabit artificially settled sperm whale carcasses off Noma Cape (approximately 250 m in depth). The former species had extracellular symbionts enclosed by the protrudent host cell membrane (the data will be published elsewhere). Enclosure by the cell membrane appears more effective to maintain extracellular symbionts than microvilli trapping. The symbionts of the latter species existed inside the host cells, as in *Bathymodiolus* mussels. These findings suggest that nutritional adaptation to the deep sea proceeded from the extracellular symbiotic state to the intracellular symbiotic state in whale carcasses. The evolutionary transition of symbiosis also supports the “Evolutionary stepping stone hypothesis”. *Benthomodiolus geikotsucola* is one of mytilid mussels that can live in the deepest sea and thus is more adaptive to abyssal waters than vent/seep bathymodioline mussels (their habitats are up to 4,000 m in depth), but maintains primitive state of symbiosis. However, some species live in sunken wood in shallower water and trap symbionts using microvilli [41].

Modioline *A. pacifica*, *A. iwaotakii* (Habe 1958), and *Idasola japonica* Habe 1976 comprised the most closely related outgroup to the bathymodioline cluster, while *A. crypta* was included in it. *Adipicola crypta* does not differ from bathymodioline mussels in the phylogenetic position and symbiotic status. Since our results indicated that monophylies of the subfamily Bathymodiolinae and the genus *Bathymodiolus* were not supported, the classification should be reevaluated. Moreover, our results showed the existence of several cryptic species, and thus more extensive morphological investigation is indispensable.

Our studies suggested that *B. brevior* was conspecific to *B. septemdierum* and might possibly be conspecific to *B. marisindicus*. Similar situations are discernible in other species. *Bathymodiolus childressi* was clearly distinguished from *B*. *mauritanicus* by the mitochondrial COI gene, but the two species did not form a separate clade based on the nuclear rDNA spacer ITS2 [24]. *Bathymodiolus boomerang* was not genetically discriminated from *B*. *heckerae* by either the COI gene or nuclear rDNA spacer ITS2 [24]. Systematics including possible synonyms should be revised, while cryptic species must be formally described.

### Evolutionary process of *Bathymodiolus* mussels

After basal trichotomous divergence of the three groups, the East Pacific subcluster diverged in Group 2. Next the Atlantic and Indo-West Pacific subclusters split (Fig. 3). However, the divergence of the three subclusters also appears trichotomous, because alliance of the latter subclusters was marginally supported. Divergence of the Atlantic and East Pacific subclusters may have been caused by the closure of the Isthmus of Panama. The rise of the Isthmus of Panama began in the middle Miocene (15.5 to 12 MYA), an island chain emerged 13 to 12 MYA, and in the late Miocene (11.5∼8 MYA) terrestrial species were able to move between North and South America [42]. Although the final closure was only accomplished by 3 to 3.5 MYA, faunal changes between the East Pacific Ocean and the Caribbean Sea had begun long before. The formation of the Isthmus of Panama has exerted profound effects on shallow-water animals since the late Miocene. Diversification of reef corals in the Caribbean, followed by an increase in carbonate-associated benthic foraminiferans, began in the late Miocene [43]. Snapping shrimps, living at depths of less than 20 m in mangrove stands and shallow waters, diverged between the East Pacific and the Caribbean 9 to 3 MYA [44]. Transisthmian divergence of shallow-water gastropods occurred 8.5 to 5.3 MYA [45]. It seems reasonable to consider that the transisthmian isolation of deep-sea animals preceded that of shallow-sea animals, although this depends on larval behavior and physiology. It is not known whether larvae of *Bathymodiolus* mussels are transported by bottom currents, but the blocking of larval transport through the closing transisthmian seaway is more likely for deep-sea animals than for shallow-water animals. The isotope composition of hydrogenous ferromanganese crusts from the western North Atlantic and the central Pacific Oceans suggests that the compositional shift around 12 MYA might have been related to the initial shallowing of the Central American Isthmus, which prevented the access of deep water from the North Atlantic [46].

It is very difficult to set unequivocal reference times. Nevertheless, we tentatively set reference times of 12 to 10 MYA for the split between the Atlantic and East Pacific subclusters. We provisionally propose here a hypothesis on the evolutionary process of *Bathymodiolus* mussels to provide a basis for discussions of the evolution of deep-sea animals.

According to the evolutionary time estimated by Thorne and Kishino's approach (Table 2), the three groups are estimated to have diverged in the Miocene, when climates changed markedly. It is generally accepted that transgression and regression concomitant with global warming and cooling lead to worldwide dispersal and diversification of sea animals. If this is the case for deep-sea animals, the ancestor of *Bathymodiolus* established worldwide distribution in the sea enlarged by transgression in the early Miocene. Subsequently, in the early to middle Miocene, diversification was caused by vicariance due to regression and plate tectonic events. Our estimate of the onset of bathymodiolin diversification (*ca*. 21 MYA) is roughly consistent with the younger estimate based on 18S rRNA data that showed that the common ancestor of modern bathymodioline vent and seep species might have lived as early as 22 MYA [47], [48]. If the ancestor of the Bathymodiolinae originated in the Miocene, it is possible that it used whale carcasses as evolutionary stepping stones for progressive adaptation from shallow to deep waters, because whale carcasses have been available since the late Eocene (*ca.* 39 MYA [49]).

**Table 2:** Sample list.

Species	Sample abbreviation	Sampling site (locality number in Fig. 1)	Depth (m)	Habitat type
**Bathymodiolinae**				
*Bathymodiolus aduloides*	AK1	Off Kikaijima Island (1)	1 451	Seep
*B. azoricus*	AZL1	Lucky Strike, Mid-Atlantic Ridge (2)	Unknown	Vent
*B. brevior* NF	BN	Mussele Valley, North Fiji Basin (3)	Unknown	Vent
*B. childressi*	ChiG1	Gulf of Mexico(4)	1 859	Seep
*B. hirtus*	HK1	Kuroshima Knoll, Off Yaeyama Islands (5)	637	Seep
*B. japonicus*	JH1	Off Hatsushima, Sagami Bay (6)	1 170–1 180	Seep
*B. marisindicus*	MK1	Kairei Field, Southern Central Indian Ridge (7)	2 443–2 454	Vent
*B. platifrons*	PH1	Off Hatsushima, Sagami Bay (6)	1 170–1 180	Seep
*B. puteoserpentis*	PUS1	Snake Pit, Mid-Atlantic Ridge (8)	3 023–3 510	Vent
*B. securiformis*	LK1	Kuroshima Knoll, Off Yaeyama Islands (5)	641	Seep
*B. septemdierum*	SM1	Myojin Knoll, Izu-Ogasawara Island-arc (9)	1 288–1 290	Vent
*B. thermophilus*	ThE1	9N East Pacific Rise (10)	2 524	Vent
Chamorro *B.* sp.	C1	South Chamorro Seamount, Mariana (11)	2 899	Seep
Eifuku *B.* sp.	EF1	Northwest Eifuku Seamount (12)	1 625	Vent
Kikaijima *B.* sp.	Kikaijima	Off Kikaijima Island (1)	1 430	Seep
Lau *B.* sp. 1	Lau1	Hine Hina, Lau Basin (13)	1 818	vent
Lau *B.* sp. 2	BR1	Hine Hina, Lau Basin (13)	1 818	Vent
*B. manusensis*	BE1	PACKMANUS Field E, Manus Basin (14)	1 627–1 629	Vent
NZ *B.* sp.	Ne1	Off New Zea land (unknown)	Unknown	Vent
Sissano *B.* sp. 1	Si2-1	Sissano, Papua New Guinea (15)	1 646	Seep
Sissano *B.* sp. 2	Si1-1	Sissano, Papua New Guinea (15)	1 881	Seep
Sissano *B.* sp. 3	Si3-3	Sissano, Papua New Guinea (15)	1 881	Seep
*Gigantidas horikoshii*	Kaikata	Kaikata Seamount (16)	486	Vent
Aitape *G.* sp.	Aitape1	Aitape, Papua New Guinea (17)	470	Seep
Ashizuri *G.* sp.	Ashizuri	Off Ashizuri Cape (18)	575	Seep
Nikko *G.* sp.	NK1	Nikko Seamount (19)	485	Vent
Sumisu *G.* sp.	Su1	Sumisu Caldera (20)	676–686	Vent
**Database**				
*B. brooksi*	*B. brooksi*WFE(DB)	West Florida Escarpment (21)	3 314	Seep
*B. heckerae*	*B. heckerae*WFE(DB)	West Florida Escarpment (21)	3 314	Seep
*B. mauritanicus*	*B. mauritanicus*(DB)	West Africa (22)	1 000–1 267	Seep
*B.* sp. East Pacific	*B. aff. thermophilus*(DB)	32S East Pacific Rise (23)	2 331	Vent
*B.* sp. NZ3	*B.* sp. NZ3(DB)	Macauley Cone (24)	200	Vent
*B. tangaroa*	*B. tangaroa*(DB)	Off Turnagain Cape, New Zea land (25)	920–1 205	Seep
*B. brevior* MT	*B. brevior*MT(DB)	Mariana Trough (26)	3 589	Vent
*Gigantidas gladius*	*Gigantidas gladius*(DB)	Rumble III (27)	300–460	Vent
*Tamu fisheri*	*Tamu fisheri*(DB)	Garden Banks (28)	546–650	Seep
**Modiolinae & Mytilinae**				
*Adipicola crypta*	ACN1	Off Noma Cape, Kagoshima (29)	225–229	Whale bone
*Adipicola iwaotakii*	AIH1	Off Nakaminato, Ibaraki (30)	490	Wood
*Adipicola pacifica*	APN1	Off Noma Cape, Kagoshima (29)	225–229	Whale bone
*Idasola japonica*	IJN1	Off Noma Cape, Kagoshima (29)	400∼425	Wood
*Benthomodiolus geikotsucola*	Tori1-1	Torishima Seamount (31)	4 051	Whale bone
*Modiolus nipponicus*	*Modiolus nipponicus*	Off Oura Harbor, Shizuika	-	Shallow
**Database**				
*Benthomodiolus lignicola*	*Benthomodiolus lignicola*(DB)	Chatham Rise (32)	826–1 174	Whale bone, Wood
*Idas macdonaldi*	*Idas macdonaldi*(DB)	Garden Banks (28)	650	Seep
*Idas washingtonia*	*Idas washingtonia*(DB)	Monterey Bay (33)	960–1 910	Whale bone, Wood, Vent

Species in Group 3 could be relics of cosmopolitans, because there are only two species, the distribution of which is restricted to the West Pacific although they have a long history (since *ca.* 8.4 MYA). Species in Group 1-1 started differentiating *ca*. 11 MYA, and the four species in the Okinawa Trough, *B. hirtus*, *B. japonicus*, *B. platifrons*, and *B. securiformis*, speciated 3.5 to 9.8 MYA, long before the formation of the Okinawa Trough (*ca.* 2 MYA). Thus, members of Group 1-1 living in the Okinawa Trough speciated elsewhere and thereafter migrated to the Okinawa Trough [15]. It is unlikely that species in Group 1-1 are also relics of cosmopolitans. They are distributed in highly isolated localities, like Japanese waters and the East and West Atlantic, without any species in intervening areas. However, our results showed that *B. platifrons* from Japanese waters was very closely related to *B. childressi* from the West Atlantic and trans-Atlantic *B. mauritanicus* (Fig. 3). It is evident that further surveys of novel vent and seep sites and genetic examination of deep-sea mussels are needed to discover cryptic members of Groups 3 and 1-1 and to elucidate the evolutionary history of *Bathymodiolus* mussels.

## Materials and Methods

### Materials and sequencing of mitochondrial genes

Specimens used in this study are listed in Table 2 and collection sites are mapped in Figs. 1 and 2. Sequencing was performed as described previously [15], [18], [20]. Since the doubly uniparental inheritance of mitochondrial DNA is known in some mytilid mussels, we have included at least five specimens, if available, of each nominal and cryptic species in our previous analyses to detect divergent and highly heterogeneous DNA sequences [15], [16], [19]. However, we used one specimen of each species, because we have not seen any evidence of doubly uniparental inheritance so far. Nevertheless, it is not plausible to represent each species by a single sequence, especially for mytilid mussels from sunken whale carcasses and wood and shallow water, although many phylogenetic studies did so. Heteroplasmy of mitochondrial DNA was shown even in *Bathymodiolus* [50].

### Phylogenetic analysis

DNA sequences were edited and aligned using DNASIS (Hitachi Software Engineering Co., Ltd., Tokyo, Japan) and MEGA 3.1 software [51], and the alignments were corrected by visual inspection for phylogenetic analysis. We used 401-bp COI and 423-bp ND4 sequences, excluding ambiguous sites. Dendrograms were constructed using the neighbor-joining (NJ) and maximum parsimony (MP) methods using PAUP*4.0 beta 10 software [52]. Genetic distances were computed using the Kimura's two-parameter method [53]. The reliability of trees was evaluated by producing 1,000 bootstrap replicates. The majority-rule consensus MP tree was constructed by conducting a heuristic search based on the 1,000 bootstrap replicates with an unweighted ts/tv ratio. The Bayesian tree was constructed using MrBayes version 3.1 software [54] based on the model evaluated by the Mrmodel test 2.2 [55]. The Monte Carlo Markov chain (MCMC) length was 5×10^6^ generations, and we sampled the chain after every 100 generations. MCMC convergence was assessed by calculating the potential scale reduction factor, and the first 1×10^4^ generations were discarded. We used *Modiolus nipponicus* (Mytilidae, Modiolinae) as an outgroup species.

Evolutionary divergence times were estimated using the relaxed molecular clock model implemented in the software Multidivtime [56], [57]. This model depends on the Maximum Likelihood topology, which was inferred with PAUP [52] using the Hasegawa-Kishino-Yano evolutionary model [58] assuming that rates across sites vary according to a discretized gamma distribution [59]. Under this relaxed molecular clock model the proportion of times from internal nodes to the ingroup root node – the root when we exclude the outgroup and then reroot the tree – are robust to time scaling [57]. Thus a preliminary run of Multidivtime without any calibration point was conducted to find an initial guess for the divergence time of the ingroup root node. The Multidivtime analysis was conducted assuming a gamma distribution with mean and standard deviation of 30MYA for the divergence time of the ingroup root node. Furthermore the splitting time between the Atlantic and East Pacific subclusters was calibrated to be between 10 and 12MYA. We then sampled 10^3^ posterior estimates of divergence times and other parameters at every 10^4^ iterations after discarding the first 10^5^ samples.
